# How to Slow down the Ticking Clock: Age-Associated Epigenetic Alterations and Related Interventions to Extend Life Span

**DOI:** 10.3390/cells11030468

**Published:** 2022-01-29

**Authors:** Anne-Marie Galow, Shahaf Peleg

**Affiliations:** 1 Institute for Genome Biology, Research Institute for Farm Animal Biology (FBN), 18196 Dummerstorf, Germany; 2 Research Group Epigenetics, Metabolism and Longevity, Research Institute for Farm Animal Biology (FBN), 18196 Dummerstorf, Germany; 3 Institute of Neuroregeneration and Neurorehabilitation of Qingdao University, Qingdao 266071, China

**Keywords:** histone modification, DNA methylation, metabolism, longevity, health span, biomarker, age-associated disorders, methylation clock, DNAm age, accelerated epigenetic aging, microbiome, single-cell sequencing, deep learning

## Abstract

Epigenetic alterations pose one major hallmark of organismal aging. Here, we provide an overview on recent findings describing the epigenetic changes that arise during aging and in related maladies such as neurodegeneration and cancer. Specifically, we focus on alterations of histone modifications and DNA methylation and illustrate the link with metabolic pathways. Age-related epigenetic, transcriptional and metabolic deregulations are highly interconnected, which renders dissociating cause and effect complicated. However, growing amounts of evidence support the notion that aging is not only accompanied by epigenetic alterations, but also at least in part induced by those. DNA methylation clocks emerged as a tool to objectively determine biological aging and turned out as a valuable source in search of factors positively and negatively impacting human life span. Moreover, specific epigenetic signatures can be used as biomarkers for age-associated disorders or even as targets for therapeutic approaches, as will be covered in this review. Finally, we summarize recent potential intervention strategies that target epigenetic mechanisms to extend healthy life span and provide an outlook on future developments in the field of longevity research.

## 1. Introduction

As of the year 2021, aging is considered both an intriguing process that research attempts to understand and a universal burden that the scientific community and the industry seek to intervene with. Currently, various theories have been put forward as to how we age, which physical alterations occur during aging and how we could substantially increase healthy life span or even maximal life span [1,2,3]. In 2013, a comprehensive review by Lopez-Otin and colleagues proposed a detailed framework incorporating nine hallmarks of aging to characterize this complex process [4]. These hallmarks comprise epigenetic alterations, telomere attrition, genomic instability, loss of proteostasis, mitochondrial dysfunction, cellular senescence, stem cell exhaustion, deregulated nutrient sensing, and altered intercellular communication. Intriguingly, these attributes are highly interconnected [5,6]. Here, we will focus on age-related epigenetic alterations and how targeting the epigenetic landscape might enable extension of life span. However, interconnections of epigenetic alterations and other hallmarks of aging will also be addressed in this review.

The term “epigenetics” was first introduced by C. Waddington, who represented cellular fate decisions during development with a ball rolling down an epigenetic landscape [7]. Thereafter, underlying epigenetic mechanisms such as histone modifications and DNA methylation were discovered at the end of the 20th century and now have a well-established role in the regulation of gene expression [8,9]. In general, histones are bound to DNA in order to compact it to accommodate the size of the nucleus [10]. This DNA-histone interaction is dynamic. The modifications of the tail domain of histones by small molecules can alter the interaction between the DNA and histone thus changing the accessibility of that specific genomic area [11]. In this way, histone tail modifications can modulate the activation, silencing or rate of transcription [9,11].

DNA methylation refers to the covalent binding of a methyl group to the fifth position of the cytosine ring (5-methylcytosine) or the sixth position of adenine (6-methyladenine) catalyzed by DNA methyltransferases (DNMTs) [12]. Methylation to 5-methylcytosine is the most prevalent DNA methylation in eukaryotes and predominantly occurs on cytosines preceding a guanine nucleotide, so-called CpG sites [13]. Moreover, methylated cytosines can be oxidized to 5-hydroxymethylcytosine by ten-eleven translocation (TET) enzymes [14]. Although there are approximately 28 million CpGs in mammals, those CpGs sites are very sparse for most of the genome while they cluster in some so-called CpG islands, which are often located at gene promoters or regulatory sequences, including enhancers. In general, 5-methylcytosine is considered to suppress gene expression by preventing binding of transcription factors to the respective promoter regions [15]. Moreover, the translocation of methyl CpG binding protein 2 to methylated CpG sites can suppress transcription by recruiting respective histone modifying enzymes to these regions [16].

**Figure 1:**
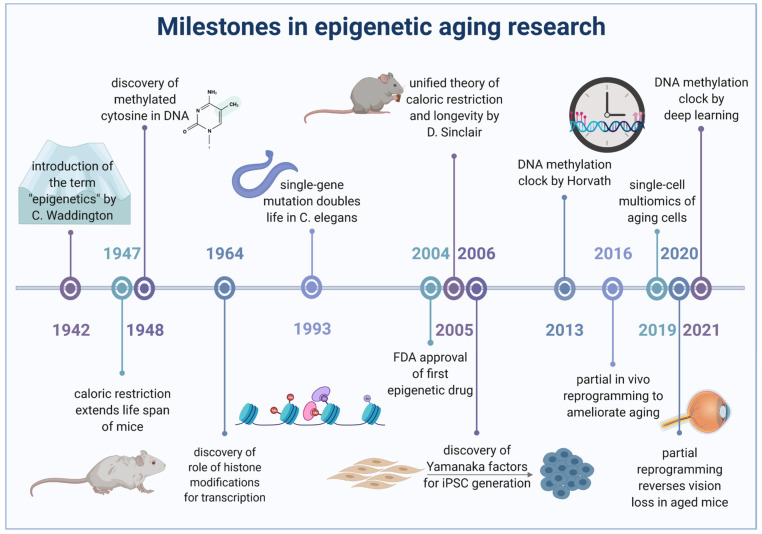
Relevant milestones in research on epigenetic aging and longevity interventions. While research in the 1950s built the foundation for epigenetic studies and in-depth analyses of longevity factors at the beginning of the new century, recent research focuses more and more on methods to directly exploit epigenetic mechanisms to prevent or even reset aging.

For a long time, aging was believed to be genetically determined [17] and research focused on specific genes and pathways that could be targeted to prolong life. Back then, the finding that a mutation in the daf2 gene, encoding the insulin-like growth factor 1, was sufficient to double the lifespan in C. elegans [18], was a huge milestone in aging research. However, with time perspectives on aging changed and it is now known that aging is not only accompanied by alterations in the epigenetic landscape, but also at least in part induced by those changes. Here, we present recent findings on epigenetic changes involving histone modifications and DNA methylation during aging and age-associated maladies such as neurodegeneration and cancer. In this regard, we also outline the emergence of DNA methylation clocks to determine biological aging. We will cover the utility of epigenetic signatures as biomarkers and the physiological implications of respective alterations. Age-associated metabolic dysregulation, which could underlie epigenetic changes, and other risk factors for age acceleration, will be described before we finally explore therapeutic interventions aiming to prevent age-associated maladies and to increase healthy life span including the emerging field of cellular reprogramming. Figure 1 illustrates some of the most relevant milestones that paved the long way from the onset of epigenetic research to the most recent intervention strategies targeting these complex mechanisms.

## 2. Age-Related Changes in Histone Modifications

The chromatin that builds up our chromosomes can be classified into two states: the open and actively transcribed euchromatin and the closed and transcriptionally inactive heterochromatin [19]. The accessibility of genomic areas within the DNA is altered via histone tail modifications, typically involving lysine acetylation, lysine methylation, arginine methylation, serine phosphorylation and others [20,21], which collectively modulate gene transcription. While some marks are associated with transcriptional silencing such as H3K9me3, H3K27me3, H4K20me2 and H3K56ac [9], other marks induce active transcription including H3K4me3, H3K36me3 and H4K16ac [9]. During aging, these modification sites are subject to changes and a global reduction in heterochromatin can be observed in various species [22,23,24]. From yeast to humans, aging is generally accompanied by a transition to more euchromatic states in specific regions that are normally heterochromatic, including telomeres and peri-centromeres [25]. Yet, distinct foci of heterochromatin may also form in context with aging thereby mediating specific changes in gene expression.

### 2.1. Alterations of Specific Histone Acetylation and Methylation Sites during Aging

In general, activating modifications increase globally while repressing modifications decrease during the process of aging. Nonetheless, the age-related pattern of histone modifications differs between individuals, tissues, and even between cells of the same tissue [26]. Several previous excellent reviews have thoroughly discussed various epigenetic marks associated with aging [4,27,28,29,30]. Illustrating various trends (decrease/increase/remodel/no change) for specific histone modifications, the main epigenetic alterations observed during aging are well summarized in Benayoun et al., 2015 [27].

More recent results showing histone acetylation changes in aging will be included here. Specific modifications such as H4K16 acetylation (H4K16ac) were demonstrated to increase with age in yeast [31] and genomic enrichment of this modification was also characterized in aged human brains [32]. Moreover, the neighboring H4K12 acetylation (H4K12ac) was also found to be increased during middle age of drosophila while experimentally reducing acetylation of H4K12ac improved the life span of the flies [33]. Similarly, H4K12ac is elevated in oocytes that were isolated from middle-aged (35–40 weeks old) female mice [34]. Both H4K16ac and H4K12ac have been shown to be altered in aged murine and human Peripheral Blood Mononuclear Cells (PBMCs) [35].

Changes in histone 3 acetylation were also observed in recent work reporting that CD4+ T Cells isolated from aged individuals had globally lower amounts of acetylated histone H3K9/14 compared to younger [36]. Mechanically, the reduction of histone acetylation levels was dependent on lower levels of miR181 leading to an overexpression of the deacetylase Sirtuin-1 (SIRT1) [36]. However, a recent report on liver histone acetylation revealed an age-associated increase of H3K9ac at regulatory regions of the cytochrome P450 2E1 gene while H3K27ac levels were stable [37]. Together with alterations of DNA methylation, this could indicate altered liver drug metabolism during aging mediated by epigenetic alterations [37].

ChIP experiments in the aged human prefrontal cortex and mouse brain revealed reduced levels of H3K27ac around the transcription start site of genes that are upregulated in aging, including genes associated with inflammation [38]. These findings are a bit surprising, as H3K27ac is usually linked to transcriptional activity [39]. Notably, age-associated histone acetylation changes are not only recognized in absolute levels, but are also manifested by altered occupancy. For example, a recent study in four mice tissues found no drastic changes in total core histone 3 levels during aging, but identified specific regions with altered H3 occupancy in aged mice, which may represent specific epigenetic remodeling [40].

Changes in histone methylation levels were also documented during aging. Recent work suggests that levels of the repressive histone mark H3K9me3 increased at heterochromatic regions and decreased at euchromatic regions in aged worms [41]. In line with the notion that decreasing histone methylation could be linked with prolonged life span, previous data showed that H3K9me3 was increased in the head of 40 days old versus 10 old male and female drosophila [23]. In contrast, the activating histone mark H3K4me3 is globally decreased in aging drosophila [23]. Moreover, another study on worms has demonstrated a role for H3K4me3 modifiers in affecting life span via transgenerational epigenetic inheritance, thus providing an important link between altered chromatin state in parents and epigenetic memory in their descendants [42].

In rats’ liver, levels of H4K20me3 have progressively increased between 30 days to 300 days and later to 450 days [43]. As H4K20me3 was demonstrated to be inversely correlated with gene expression in senescent cells [44], this might reflect a mechanism to compensate for the age-related increase in gene expression. Importantly, aberrant histone modifications have been linked with age-associated cryptic transcription. Previous work proposed that loss of H3K36me3 and replacement by general histone acetylation [45] in aged yeast is associated with excessive open chromatin and cryptic transcription [46], where RNA polymerase II can incorrectly initiate transcription. A reduction of H3K36me3 was also observed in 40 days old flies compared with 10 days old [23]. In line with these results, recent data confirmed the presence of cryptic transcription, due to age-associated epigenetic alterations also in mammals [47]. Hematopoietic stem cells isolated from 24 months old mice showed decreased distribution of the repressive mark H3K36me3 in gene bodies, compared to 4 months old mice [47]. Intriguingly, the authors conclude that in specific cryptic transcription start sites, this modification is replaced by increased H3K4me1, H3K4me3 and H3K27ac histone marks which results in an activation of cryptic sites [47]. While the authors concede that it remains unclear how increased cryptic transcription is linked to aging, more work is needed to establish such a link. One aspect is that cryptic transcription may severely interfere with normal transcriptional regulation during aging.

It is worth mentioning that the quantification of histone modifications by antibodies may yield inaccurate results, and perhaps even conflicting results, as these antibodies cross react with off-target modifications and also show higher affinity towards poly-modificated histones [48,49]. For this reason, we observe a transition to mass spectrometry based measurements for the quantification of histone modifications, including combinatorial analysis [33,50,51,52,53].

For example, using such an extensive mass spectrometry based analysis comparing young and old mice, Schwörer et al. have characterized an altered the epigenetic profile in quiescent muscle stem cells and activated (post muscle injury) muscle stem cells [54]. Specifically, quiescent aged stem cells show increased levels of histone modifications linked to repressive histone marks and decreased levels of histone modifications typically associated with active gene transcription [54]. Notably, following muscle injury and activation of the stem cells, the authors observed opposite responses for many histone 4 acetylation sites between the young and aged mice. Such altered epigenetic stress response has been proposed to contribute to overall decline in stem cell function [54].

Evidently, histone modifications were shown to both increase and decrease during aging, and that the nature of such alterations may rely on the organism, tissue, cell type or the gender in question [27]. Currently, no uniform histone modification alterations can be concluded as an epigenetic signature of aging, thus the view has prevailed that such modifications are generally ‘remodeled’ during aging [30]. Lastly, less is known and characterized about other histone modifications such as phosphorylation, acylation, and crotonylation in connection to aging and age-associated maladies. Future research might uncover further adaptation and regulation mechanisms relying on those modifications.

### 2.2. Metabolic Regulation of Histone Modifications during Aging

The connection between metabolic activity and modification of histones is well documented [5,21,55,56,57]. Metabolites such as acetyl-CoA, ATP, S-adenosyl methionine (SAM) and others are used as precursors to acetylate, phosphorylate and methylate histones, respectively [21,55,58], while metabolites such as NAD+ act as cofactors for the deacetylases sirtuin [59]. Notably, the connectivity of metabolism and epigenetics is bi-directional [6,56,60,61]. As metabolic activity influences the abundance of metabolites that are available for modifying histones and DNA, so does epigenetic regulation modulate the expression levels of metabolic enzymes and therefore overall metabolic rates [4,62,63,64]. For example, in flies, aging is accompanied by an increase of H3K27me3 linked to a consequential reduction of glycolytic genes [65]. These metabolic changes exert negative effects on life span, while reduction of H3K27me3 promotes glycolysis and healthy life span [65].

In general, metabolism is thought to be deregulated during aging, although in what manner is still under debate [4,62,66]. In particular, metabolic pathways that are dependent on mitochondrial function such as oxidative phosphorylation [67] and acetyl-CoA metabolism [68] appear affected by the aging process. It has been previously established that histone modifications are impacted by such age-associated metabolic alterations [56]. For example, aging is accompanied by a chronic and subtle increase in the consumption of NAD+ with the synthesis rate being relatively constant [69]. Additionally, levels of NAD+ can be depleted as a result of age-associated accumulation of oxidative nuclear damage [70,71]. The reduced availability of NAD+ causes a decreased activity of the NAD-dependent histone deacetylase SIRT1, thereby fostering increased histone acetylation [70,71].

NAD+ and glucose fuel the pyruvate dehydrogenase complex for acetyl-CoA and NADH production, but with age this complex becomes increasingly repressed by phosphorylation [72,73]. A subsequent drop in acetyl-CoA synthesis can impair histone acetylation [74]. An age-associated decrease in acetyl-CoA levels was suggested in the cortex of senescence-accelerated prone SAMP8 mice [75]. In addition, compared with 2 days old mice, 2 years old mice showed decreased acetyl-CoA signaling in cardiac stem cells [76]. Interestingly, acetyl-CoA synthesis was suggested to regulate lifespan in yeast [77]. However, the actual levels of acetyl-CoA during aging were not directly determined, and histone acetylation levels were not changed between 1 and 3 days old wild type yeast, rendering the downstream mechanism uncertain [77].

A clearer mechanism was revealed in aged mesenchymal stem cells, which demonstrate a reduced expression of citrate carrier that results in less cytoplasmic/nuclear levels of acetyl-CoA [78]. The lack of acetyl-CoA in turn causes a hypo-acetylation of histones impacting the osteogenic potential of these aged cells [78]. Another example was found in mice, where age-associated memory impairment has been linked to a lack of increased hippocampal citrate levels in response to memory stimulus in the older mice [79]. In view of the findings in mesenchymal stem cells, this is potentially attributable to a hampered increase of histone acetylation. This in turn might impact the activation of the transcriptional machinery needed for the formation of new memories [79].

However, other studies demonstrated an age-associated increase in acetyl-CoA levels. For example, studying global metabolic alterations in mouse brain hippocampus, Dong et al. observed a 2-fold increase in acetyl-CoA levels as a result of a global upregulation in fatty acid metabolism in aging mice of both genders [80]. This increase was even more pronounced when Alzheimer’s Disease-like pathologies were induced [80]. Increased acetyl-CoA levels were also detected in drosophila during mid-life [33]. Accordingly, histone acetylation was increased at specific acetylation sites in aged flies. Nonetheless, that study did not quantify acetyl-CoA levels in older flies that displayed lower mitochondrial activity [33].

**Figure 2:**
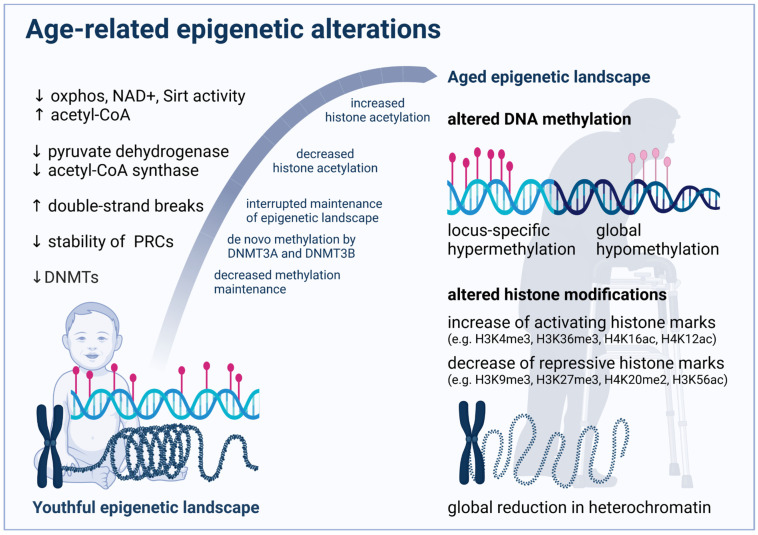
Age-related alterations and potential underlying mechanisms. Aging induces a number of metabolic and functional changes that result in a modified activity of epigenetic enzymes, eventually causing the altered epigenetic landscape of aged organisms exemplified here. However, it is not possible to deduce a general road map for epigenetic aging, as individual alterations can be specific for species, genders, tissues and cell types. Moreover, the complex interplay of the diverse regulation mechanisms is still poorly understood and likely changes at various stages throughout life.

The above-mentioned partially counteracting regulation mechanisms and contradicting data point again at the complexity in age-related histone modifications and illustrate how alterations may depend on the specific organism, tissue and cell type as well as developmental stage. Figure 2 summarizes major age-related epigenetic alterations and some potential mechanisms as outlined above.

## 3. Age-Related Changes in DNA Methylation

Throughout the aging process, DNA methylation (DNAm) levels change unevenly, demonstrating global hypomethylation and locus-specific hypermethylation [81]. Underlying mechanisms will be elucidated in the following sections and are indicated in Figure 2. Moreover, age-associated changes at some CpG islands occur relatively consistently between individuals, which motivated several groups to develop so-called epigenetic clocks to measure the chronological age of donors based on methylation data of cells, tissues or organs [82].

### 3.1. Epigenetic Clocks as Age Estimators

DNAm based epigenetic clocks are basically generated by using unsupervised machine learning methods to regress chronological age on CpGs of a training data set. A penalized regression model allows for the automatic selection of a specific set of CpGs relevant for age prediction. Based on this CpG set, the corresponding mathematical algorithm can be used to predict the chronological age in any other dataset. For this approach, array-based methods for detection of methylation sites predominate the field, although they cover only 3% of CpG sites at maximum for the most recent arrays [83]. Other methods, such as whole-genome bisulfite sequencing, cover all CpG sites, but costs for the high-depth next-generation sequencing render array-based methods are more economical [83].

Interestingly, individual CpGs can have only negligible correlation with chronological age. However, in sum, the accuracy of these clocks can be remarkably high. The two most validated clocks of Hannum et al. [84] and Horvath et al. [85] achieve correlation coefficients > 0.9 and average errors of less than five years. Based on data from more than 30 different tissues/cell types originating from newborn to adults, the epigenetic clock of Horvath et al. was the first comprehensive multi-tissue DNAm age estimator. As such, it was demonstrated to be applicable in a wide range of DNA sources and ages from prenatal to centenarian.

The number of CpG sites that were used for age estimation varies from more than thousand [86] to as low as one CpG in the *ELOVL2* gene [87], but evidence accumulates that larger sets are less error-prone and more robust when applied in different tissues [88]. While many epigenetic clocks were developed to accurately predict the chronological age, some were trained to mirror biological age and predict subsequent all-cause mortality. By additionally involving nine age-related clinical markers in the supervised machine learning process, Levine et al. developed a clock named DNAm PhenoAge that outperformed former epigenetic clocks in regard to life span prediction and its association with age-related health conditions [89]. Applying a similar strategy, Lu et al. based their CpG selection on chronological age, sex, and clinical markers for mortality, developing the so-called GrimAge clock [86]. An overview of all major epigenetic clocks in humans and mice is given in a recent review [90]. Of note, data exploration applying several existing epigenetic clocks has been facilitated for a broader user community by the freely available software package “methylclock” [91].

Age-associated changes in methylation levels were not only identified in humans and mice, but also in other mammalian species where the rate of change correlates with species lifespan [92]. Moreover, an epigenetic clock designed exclusively on ribosomal DNA methylation was demonstrated to be evolutionary conserved across several species and to respond to genetic and environmental interventions modulating life span [93]. Pan-mammalian clocks like this can provide useful biomarkers for the exploration of human age-related conditions in laboratory animal models. Moreover, the objective determination of biological age in these models facilitates the screening for compounds that can increase human health span by slowing or reversing the aging process. However, there are still gaps in the current understanding of the physiological relevance and underlying mechanics of epigenetic clocks that should be closed in the future to fully unleash the epidemiological potential of this novel biomarker for aging [94].

Such gaps in our understanding become apparent in a prominent animal model for aging research, the naked mole rat. Although not displaying an age-related increase in mortality rates and thereby deemed as non-aging, the naked mole rat was recently shown to undergo epigenetic aging as determined by methylation clocks [95]. This contradiction might imply that epigenetic changes are in fact not causative for any functional consequences of the aging process. However, the mortality rate cannot be considered as a direct representation of aging and while Horvath et al. demonstrated slower epigenetic aging in breeding queens, it was shown before that breeding animals also survive longer [96] thus underpinning a physiological relevance of epigenetic alterations. The discrepancy of epigenetic and phenotypic aging might result from efficient compensatory mechanisms to counteract age-related changes that would normally impair health and life span. For example, the naked mole rat displays high mitochondrial oxidative stress levels and does accumulate oxidative damage, yet high levels of chaperons and the proteasomal machinery are suggested to allow for an extraordinary robust proteostasis maintenance, which in turn permits the long life span [97].

### 3.2. Physiological Relevance of DNA Methylation and Epigenetic Age

Fluctuations in DNAm levels are a consequence but might also be a cause of aging. To advance towards the ultimate goals of a prolonged life span and the reversal of age-related phenotypes, it will be crucial to understand the mechanisms underlying observed associations. A first step along this way was the realization that epigenetic aging is tightly connected to the process of organismal development. The rate of methylation changes in CpGs underlying epigenetic clocks depends on the stage of life [81,85]. For example, employing the Horvath clock, this rate is approximately 24 times faster in young children than in adolescents after puberty [81,85]. Moreover, different cohort studies demonstrated that an increased rate of methylation changes is associated with more rapid pubertal development, as well as higher average weight and height [98,99,100]. Considering the function of genes in proximity to age-related methylation sites, evidence for a correlation of epigenetic aging and developmental processes consolidates. Affected genes are involved in proliferation and growth of cells as well as death and survival pathways, thereby regulating the development of tissues and the whole organism [85].

On the other hand, recent work demonstrated that accumulation of non-mutagenic double-strand breaks (DBS) results in an acceleration of age-related changes comprising physiological, cognitive, and molecular features as well as the epigenetic clock [101]. In transgenic “ICE” (inducible changes to the epigenome) mice, DBS induces the frequent recruitment of chromatin modifiers to sites that demand repair. Consequently, these modifiers are less available for maintenance of the epigenetic landscape eventually leading to the aberrant gene expression profiles seen in aged individuals [102]. In particular, with age regions of euchromatin were shown to lose the active H3K27ac mark, whereas regions of heterochromatin lose the repressive H3K27me3 mark resulting in a “flattened” epigenetic landscape [102]. Taken together, these studies implicate that epigenetic drift is a cause of aging rather than the driving force.

Interestingly, CpG sites that become hypermethylated with age are preferentially located at bivalent chromatin domain promoters simultaneously regulated by repressing and enhancing histone modifications [103]. These bivalent domains confer more dynamic cell fate decisions and are crucial for stem cell plasticity. Many of these bivalent sites are targeted by polycomb repressive complex (PRC) proteins such as Ezh2 [104,105]. PRCs normally exert repressive functions and mediate cell-fate decisions especially during embryonic development [106,107].

Several studies revealed substantial crosstalk between DNA methylation and PRC recruitment and vice versa. For example, within the PRC Ezh2 interacts with DNMTs and directly mediates promoter methylation of Ezh2-target genes [108]. On the other hand, loss of DNA methylation resulted in *de novo* recruitment of PRC proteins to the unmethylated CpG-rich sequences in murine Dnmt3a/b^−/−^ embryonic stem cells [109]. In somatic cells, inhibition of DNMTs either genetically or pharmacologically by 5-aza-2’-deoxycytidine induced similar effects demonstrating H3K27me3 redistribution to many genomic regions that are normally highly DNA methylated [110].

How PRCs recognize their target sites remains poorly understood, however, one mechanism to recruit PRCs to non-methylated DNA involves the protein KDM2B [111]. For epigenetic aging, Jung and Pfeifer concluded a competitive model in which unmethylated DNA regions are initially guarded by PRC1 and PRC2 complexes while age-related destabilization of the PRCs facilitates the access for *de novo* DNA methylation by DNMT3A and DNMT3B, subsequently resulting in the age-related hypermethylation patterns [112].

A specific example for such an interplay of DNA methylation and histone modifications can be assumed in the pathology of Alzheimer’s disease (AD). Several studies demonstrated hypermethylation in the *ANK1* gene in brain tissue of AD patients [113,114]. A recent study reports that this is accompanied by a decrease in H3K4me3, a marker of active gene transcription [115], suggesting that both epigenetic modifications concordantly cause a reduced gene activity. A study demonstrating that siRNA-mediated depletion of MLL/COMPASS, the complex that is responsible for the trimethylation of H3K4, results in hypermethylation of CpG islands [116] supports the idea that both epigenetic mechanisms are functionally interrelated.

### 3.3. Interrelations of DNA Methylation and Other Hallmarks of Aging

Besides epigenetic alterations, López-Otín concluded eight further hallmarks of aging, namely genomic instability, telomere attrition, loss of proteostasis, deregulated nutrient sensing, mitochondrial dysfunction, cellular and immunosenescence, stem cell exhaustion, and altered intercellular communication [4]. It is challenging to dissect their interrelations and relative contributions to aging. However, at least for some hallmarks there are indications that shed some light on their complex interplay that we will present here.

During aging, a gradual loss of proteostasis can be observed in many species [117] and some long-lived species are reported to have particularly stable proteomes [118]. Proteostasis relies on chaperones and two proteolytic systems, the lysosome-autophagy and ubiquitin-proteasome systems. With increasing age some lysosome-autophagy-related genes get hypermethylated via DNMTs and subsequently silenced as shown for the promoter regions of *Atg5* and *Lc3* in aged mice [119]. The suppressed expression of these and other genes essential for autophagosome biogenesis such as MAP1LC3 [120] and LAMP2 [121] disrupts the completion of autophagosomes and eventually results in autophagy failure, one of the major symptoms of aging.

Besides monocytes and macrophages, other immune cells are heavily affected by aging. Age-related DNA methylation changes impact levels of cytokines and proportions of immune cell types eventually resulting in an overall decline in immunocompetence referred to as immunosenescence. For example, in human T-cells age-related changes in DNA methylation were correlated with impaired T-cell mediated immune response [122]. Another study reported age-related hypermethylation in the *Klf14* promoter [123]. *Klf14* is involved in immune cell differentiation via the repression of *Foxp3* demonstrating a clear link between DNA methylation changes and immunosenescence. On the other side, the *Foxp3* enhancer was shown to be hypomethylated in aged mice, which was associated with higher Treg number and activity, thereby suppressing T-cell responses and further contributing to immune senescence [124].

Cellular senescence is markedly influenced by telomere attrition, however, the applicability of telomere length as biomarker for aging is recently challenged [125]. An unsatisfactory correlation of telomere length and biological age might reflect the finding that cellular senescence occurs only when telomeres reach a critical short length [126]. However, a correlation of the pace of telomere shortening and a species life span was demonstrated, stressing the relevance of this factor as another hallmark of aging [127]. Surprisingly, telomere attrition induced cellular senescence and epigenetic aging were found to be even negatively correlated. In leukocytes, single-nucleotide polymorphism variants of the telomerase reverse transcriptase gene (TERT) that are associated with longer telomeres and consequently later onset of senescence paradoxically show epigenetic age acceleration according to the Horvath clock [128]. In line with this finding, epigenetic age was increased in TERT transfected fibroblasts [129]. It is speculated that experimentally induced constant TERT expression could interfere with epigenetic maintenance, thereby accelerating epigenetic aging [130,131]. However, cellular senescence and cellular aging are generally considered distinct phenomena [132] and senescence-related methylation changes occur in different CpG sites than age-related changes [133].

In stem cells, senescence and the age-dependent decline of self-renewal capacity result in a deficiency referred to as stem cell exhaustion. Age-related methylation changes in stem cells can affect both the self-renewal capacity [134] and the differentiation capacity [135]. In elderly muscles, aging was linked to an increased methylation of a regulator of muscle stem cell quiescence (SPRY1) [136]. The consequent suppression of SPRY1 impairs the self-renewal capacity of muscle stem cells thereby limiting the regenerative potential in the elderly [136]. The relevance of DNA methylation for muscle cell regeneration was confirmed in another study demonstrating a severely decreased regeneration capacity after muscle injury in DNMT3A knock-out mice [134]. In hematopoietic stem cells (HSCs), however, transcription factor binding sites associated with HSC maintenance are reported to be hypomethylated with age, whereas genes associated with differentiation become hypermethylated. Further changes in histone modifications and a downregulation of genes encoding DNMTs and TET enzymes consequently shift aged HSCs toward self-renewal at the expense of differentiation [137]. In line with these findings, conditional inactivation of DNMT3A was shown to skew divisions toward self-renewal due to losses of DNA methylation at key regulatory regions including hematopoietic regulators such as Gata2 [138]. Similar to the phenotype of aged HSCs, the attained immortality was accompanied by a loss of differentiation capacity [138].

Mitochondrial dysfunction and oxidative stress are concomitants of aging that were mainly attributed to somatic mutations in mitochondrial DNA. However, in a study by Hashizume et al., age-associated mitochondrial respiration defects in fibroblasts from elderly subjects could be corrected by reprogramming the cells into induced pluripotent stem cells (iPSCs), suggesting that this age-associated phenotype is epigenetically regulated and not genetically manifested [139]. Microarray screening demonstrated a link between the aged phenotype and the epigenetic downregulation of genes involved in glycine production such as Glycine Acetyltransferase and Serine Hydroxymethyltransferase 2 [139]. Serine Hydroxymethyltransferase 2 is coupled to the folate and methionine cycle that yield SAM from homocysteine [140]. Since SAM acts as methyl group donor for the transferase activity of DNMTs it bridges mitochondrial glycine synthesis and DNA methylation. Moreover, the DNMT inhibitor 5-azacytidine was reported to restore mitochondrial function in aged mesenchymal stem cells evidenced by reduced accumulation of reactive oxygen species (ROS) and nitric oxide [141]. Mitochondrial function and DNA methylation are linked by the transcription factors NRF1 and PGC1-α that can enhance DNMT1 expression upon activation by oxidative stress and hypoxia. Translocated into mitochondria, DNMT1 was shown to differentially modify the transcription of mitochondrially encoded genes [142]. Moreover, a high proportion of noncoding regions of mitochondrial DNA was reported to be hypomethylated in replicative senescent cells potentially due to a downregulation of mitochondrial DNMT, thereby linking DNA methylation, senescence and mitochondrial function [143].

## 4. Epigenetic Changes as Biomarker in Age-Related Diseases

Deregulations of histone modifications and errors in DNA methylation can accumulate during aging, thus increasing the risk for age-related pathologies such as diabetes, neurodegenerative disorders, cardiovascular diseases, and cancer. Based on epigenetic clocks and underlying differentially methylated regions, efforts are in progress to develop epigenetic based biomarkers for these conditions. Ultimately, the availability of prognostic biomarkers might enable early diagnosis and treatment of presymptomatic patients thereby extending health and life span.

### 4.1. Diabetes

The increased life span in humans is accompanied by a rising prevalence of diabetes in the older population. Tests for screening and diagnosis of diabetes are well established. However, DNAm based biomarkers might serve as prognostic tools in the future. The role of epigenetics in type 1 [144] and type 2 [145] diabetes have recently been reviewed by others. Here, we concentrate on findings most relevant for potential applicability in clinical settings. 

In type 2 diabetes, β-cells dysfunction and insulin resistance result in abnormally elevated blood glucose levels. Longitudinally following a healthy volunteer for three years, Chen et al. found changes in DNA methylation in PBMCs three month before elevated glucose levels have manifested [146]. To identify CpGs predictive for the risk of developing type 2 diabetes, Toperoff & Aran et al. employed a pool-based, genome-scale screening [147]. They found a CpG site in the fat mass and obesity-associated (*FTO*) gene as hypomethylated yet before the onset of diabetes and confirmed this finding in a subsequent prospective study [147]. In a large epigenome-wide association study, 62 type 2 diabetes associated loci were found and used to calculate a weighted “Methylation Risk Score” that outperformed conventional risk factors such as obesity, fasting glucose, and hyperinsulinemia in prediction power for the onset of type 2 diabetes [148]. Similarly, a methylation score based on five markers located in *ABCG1, PHOSPHO1, SOCS3, SREBF1*, and *TXNIP* could predict future type 2 diabetes incidence independent of established risk factors [149]. The association of *ABCG1, SREBF1*, and *TXNIP* with the incidence of type 2 diabetes could be reaffirmed in an independent replication study, thus rendering these factors promising for clinical use as biomarkers [150].

Of particular interest, the prevalence of type 2 diabetes during aging is at least partially linked with increased inflammation [151] and several inflammatory genes are regulated by epigenetic mechanisms. For example, in macrophages of diabetic mice an increase of the major inflammatory regulator Nuclear factor κ-B (NF-κB) was shown to be induced by the methyltransferase SET7/9, mediated by increased levels of H3K4me [152]. Another study in diabetic mice reports that the increased expression of inflammatory factors such as IL6 correlates with decreased levels of the repressive mark H3K9me3 in the promoter of these genes [153]. Mechanistically, the authors suggest that this lower abundance is mediated by reduced methyltransferase Suv39h1 levels. When normal human vascular smooth muscle cells were cultured in high glucose the same decrease in H3K9me3 and increase in inflammatory gene expression was observed [153]. A general overview on the link between epigenetic alterations and diabetes is further discussed by Ling and Groop [154].

### 4.2. Alzheimer’s Disease

At present, a definitive diagnosis of Alzheimer’s disease (AD) can only be achieved by neuropathological examination of the patient’s brain tissue after death. Hence, the identification of novel clinical biomarkers for early diagnosis of AD is urgently needed. Although a recent study of DNAm-based measures in the Lothian Birth Cohort 1921 could not find a positive correlation between increased epigenetic age compared to chronological age (epigenetic age acceleration) and dementia risk [155], correlations were found for neuropathological markers of AD, the most common form of dementia. Epigenetic age acceleration in the dorsolateral prefrontal cortex is reported to be associated with AD related neuropathological markers such as diffuse plaques, neuritic plaques, and amyloid load as well as impairments of general cognitive capacities and memory [156]. Moreover, differentially methylated regions affecting the expression of *ANK1, CDH23, DIP2A, RHBDF2, RPL13, RNF34, SERPINF1* and *SERPINF2* were shown to be associated with AD while being already recognizable in presymptomatic subjects [114].

Obviously, blood is much more accessible for diagnostic purposes. However, differentially methylated regions in blood were shown to be distinct to those identified in the brain. For example, although *ANK1* hypermethylation in brain tissue was validated as one of the most robust molecular markers for AD, its differential methylation was not detected in blood. In blood, modifications were observed in proximity to genes such as *DAPK1, GAS1*, and *NDUFS5* [113]. In a genome-wide screening of blood DNAm levels in amnestic mild cognitive impairment (aMCI) and AD patients, hypomethylation in the *NCAPH2/LMF2* promoter region and hypermethylation in the *COASY* and *SPINT1* gene promoter regions were identified [157] and proposed as potential diagnostic biomarkers for AD and aMCI.

Notably, these studies were of retrospective nature and thus not sufficient to confirm the predictive power of identified biomarkers. The German Study on Ageing, Cognition, and Dementia (AgeCoDe), however, is a prospective study focusing on identification of risk factors and predictors of cognitive decline and dementia in elderly patients that might serve as a valuable source of data. Between 2003 and 2004, more than 3000 volunteers that did not suffer from dementia at that time were recruited and since then interviewed at regular intervals. In 2019, Lardenoije et al. investigated the DNA methylation of 55 subjects that developed AD dementia in the course of the study and identified several differentially methylated regions in their blood at baseline [158]. Interestingly, only one of these regions, close to *GLIPR1L2*, showed hypermethylation both at baseline and after transition to AD at follow-up, stressing the limitations of retrospective studies in affected cohorts to identify prognostic biomarkers.

Considering histone modifications, a comparison of the brains of healthy humans and AD patients showed a lower total number of H4K16ac peaks and a redistribution of this activating histone mark [32]. Moreover, reduced levels of H4K12ac were linked to an over-expression of Lysine deacetylase (KDAC) 2 in a murine neurodegeneration model, which was also observed in AD patients [159]. Of note, administration of RGFP-966, a selective Histone Deacetylase 3 inhibitor, caused increased H4K12 acetylation and resulted in an improvement in several memory tasks in an AD mouse model [160]. In fact, treatment with KDAC inhibitors was shown to improve cognitive function in a number of studies on aged animals and neurodegenerative mouse models [79,161,162,163,164,165,166]. While the discovery of appropriate biomarkers for early AD diagnosis is still challenging, a KDAC inhibitor based therapeutic approach to mitigate the burden of age-related neurological impairments might soon be in hand.

### 4.3. Cardiovascular Diseases

Cardiovascular diseases (CVDs) were linked with epigenetic age acceleration based on different epigenetic clocks [167,168] and several studies were conducted to assess the association of specific methylation sites with the risk for CVDs. In 2017, a systematic review considering global methylation, candidate-gene, and epigenome-wide association studies concluded that global DNA methylation is not correlated with the onset of CVDs, while hypermethylation in *ESRα, ABCG1* and *FOXP3* and hypomethylation in *IL-6* are associated with coronary heart disease. CVD associated genes identified in epigenome-wide association studies are functionally involved in obesity, lipid and carbohydrate metabolism, as well as inflammation processes [169]. In line with these findings, Infante et al. detected increased methylation levels in genes relevant for cholesterol metabolism such as *LDLR* in peripheral blood of coronary heart disease patients compared to healthy controls. Based on methylated DNA immunoprecipitation and image data of Cardiac Computed Tomography they found that increased *LDLR* promoter methylation was correlated with calcified plaque volume and total plaque burden. Moreover, increased ABCA1 and SREBF2 expression were proposed as predictors of coronary heart disease and severity of disease, respectively [170].

For atherosclerosis, DNA methylation was demonstrated to mainly affect genes concerning functions of endothelial cells (e.g., *HOXB3, EGFR*), smooth muscle cells (e.g., *CALD1, RPTOR*), and macrophages (e.g., *PDGFD*) while being associated with oxidative stress and inflammation [171]. Aberrant DNAm profiles of atherosclerotic lesions are reported to become more frequent with histological grade. With lesion progression, a range of CpGs affecting genes implicated in macrophage biology and inflammation drifted towards hypermethylation in the aorta [172]. Differential methylation in some genes, namely *C1QL4*, *CTNNA3* and *IMMT*, was shown before in peripheral blood samples [173]. However, just like in Alzheimer’s disease, there is a considerable inconsistency in methylomes of the easily accessible peripheral blood cells and the actually affected tissue. Moreover, a relatively low study-to-study reproducibility impedes the successful discovery of suitable prognostic biomarkers [174]. A recent prospective multi-cohort study including a discovery cohort (the Strong Heart Study) and four additional cohorts, confirmed an association between certain blood DNAm signatures and coronary heart diseases. Yet, of the 505 differentially methylated CpG sites identified in the Strong Heart Study, only 33 were commonly selected in the other cohorts and some of them demonstrated associations in opposite directions across cohorts [175]. Lately, the focus shifted towards hydroxymethylation as a diagnostic parameter for coronary atherosclerosis. Increased 5-hydroxymethylcytosine levels in PBMCs were linked to higher severity of carotid and coronary atherosclerosis [176] and thus identified as a risk factor, accordingly rendering TET2 a potential target for atherosclerosis treatment [177].

### 4.4. Cancer

Along with accumulation of genetic mutations, epigenetic alterations and transcriptional deregulation are causally linked with cancer [178,179]. In fact, mutations in DNAm related epigenetic modifiers were identified in 5–21% of patients from The Cancer Genome Atlas (TCGA) for 11 analyzed cancer types [180]. Hypomethylation in tumorous tissue was reported for several oncogenes such as *HOXC10* [181], *uPA* [182] and *CT45* [183]. On the other hand, cancer-associated hypermethylation was found in promoters of genes involved in DNA repair (e.g., *BRCA1* [184], *MLH1* [185], *MGMT* [186]), apoptosis (e.g., p53 target genes *DAPK1* [187] and *CASP8* [188]), and immune response (e.g., *CXCL9, CXCL10* [189], *NKG2D* [190], and *MHC1* related genes [191]). 

To diagnose cancer of unknown primary, several tools were developed to identify the cell-of-origin based on methylation assays. In a multicenter retrospective analysis, the diagnostic tool EPICUP for instance correctly classified the origin of 87% of analyzed tumor samples representing 38 tumor types [192]. However, as with the former age-related diseases, potential biomarkers should ideally be detectable in body fluids. Indeed, liquid biopsies from blood, urine, or stool can contain circulating tumor cells and tumor DNA (ctDNA), microvesicles such as exosomes or other informative molecules linked to tumorigenesis. ctDNA is the cancer-originating component of cell free DNA (cfDNA), which is frequently used for DNAm analysis [193]. Based on the comprehensive data provided by TCGA and the Gene Expression Omnibus, cancer type-specific DNAm-based markers were identified and used to computationally infer cell-type composition in cfDNA samples of patients [194,195,196]. A comprehensive overview on cell-free DNAm-based methods and their applications in oncology was recently published [197]. Spin out companies such as EarlyDiagnostics translate these findings. Moreover, the company Grail (https://grail.com/, accessed on 3 January 2022) developed a multi-cancer early detection test for cancer screening using DNAm patterns in blood. For this, more than 180,000 participants in North America and the United Kingdom have been enrolled in four observational, clinical, cohort studies with longitudinal follow-up (CCGA-NCT02889978; STRIVE-NCT03085888; SUMMIT-NCT03934866; PATHFINDER-NCT04241796). With a high sensitivity for stage I-III cancers and a specificity around 95%, Grail demonstrated that methylome sequencing of cfDNA can outperform approaches relying on somatic mutations for cancer diagnosis [198,199]. By now, several companies developed commercial tests that are approved for clinical use as reviewed in Locke et al. [200].

Epigenetic features are not only employed for diagnostic purposes but might also serve as prognostic factors to predict the outcome of therapeutic interventions. For example, the cancer-associated hypermethylation of the *MGMT* promoter and the consequent downregulation of the encoded DNA alkylation repair protein sensitizes tumor tissues for alkylating chemotherapeutics and was associated with tumor regression consequently extending overall and disease-free survival in glioma patients [201]. Thus, *MGMT* promoter methylation serves as a predictor of tumor responsiveness to alkylating agents. More generally, in a screening study of 58 human cancer cell lines from the National Cancer Institute drug-screening panel, a strong correlation of hypermethylation and subsequent gene silencing of the p53 homologue p73 and the sensitivity to alkylating chemotherapeutics was suggested [202]. There are several more DNAm sites that were demonstrated to be correlated with chemotherapy efficacy in a variety of cancers e.g., *CHFR* and *MLH1* in gastric cancer [203], *FANCF* in ovarian cancer [204], *SFN* [205], and *IGFBP3* [206] in lung cancer, as well as *MT1E* in melanoma [207], demonstrating the potential applicability of epigenetic markers not only to predict the therapy outcome but also to choose an adequate therapy approach.

In human melanoma, neoplastic clones negative for Cancer/testis antigens (CTA) such as MAGE-A3 hamper the effectiveness of CTA-based vaccine therapy [208]. The heterogeneous expression of MAGE-A3 has been shown to be correlated with its promoter methylation status. Notably, the DNMT inhibitor 5-aza-2’-deoxycytidine could reinduce MAGE-A3 expression in CTA-negative clones thereby sensitizing them to immunotherapy [208]. The global demethylation induced by DNMT inhibitors often results in re-expression of genes with tumor suppressive actions such as induction of apoptosis, cell cycle arrest, or immune response. Accordingly, DNMT inhibiting agents were reported to increase the susceptibility to immunotherapy in several cancer types [209,210,211]. Decitabine (5-aza-2´-deoxycytidine) and Azacytidine (5-azacytosine) demonstrated significant benefits in clinical trials and have been approved by the FDA for the treatment of myelodysplastic syndrome [212,213].

For histone modifications, early work has identified both reduced H4K16ac and H4K12 tri-methylation as a hallmark for cancer [214]. Several excellent extensive reviews discuss the growing evidence of histone modifications changes in cancer [215,216,217,218,219]. Other works have established a link between altered expression of lysine acetyltransferase or lysine deacetylases and cancer [220]. Accordingly, several KDAC inhibitors are studied as therapeutic avenues to treat cancer and are currently tested in various phases of clinical trials [221,222,223]. However, it is noteworthy that KDAC inhibitors such as butyrate also serve as metabolites that can be used by cancer cells [224], thereby directly affecting their metabolic activity [225] which may be independent of histone acetylation. Further, KDAC treatment may also target non-histone proteins [226]. As such, we should approach the mechanistic therapeutic benefits of KDAC inhibitors in cancer with caution.

In summary, DNAm based markers can be utilized for primary diagnosis and prognosis as well as choice of therapy approach and disease monitoring, while some epigenetic modifying agents are tested or already approved for cancer treatment thereby counteracting one deleterious concomitant of aging.

## 5. Risk Factors Accelerating Epigenetic Aging

**Figure 3:**
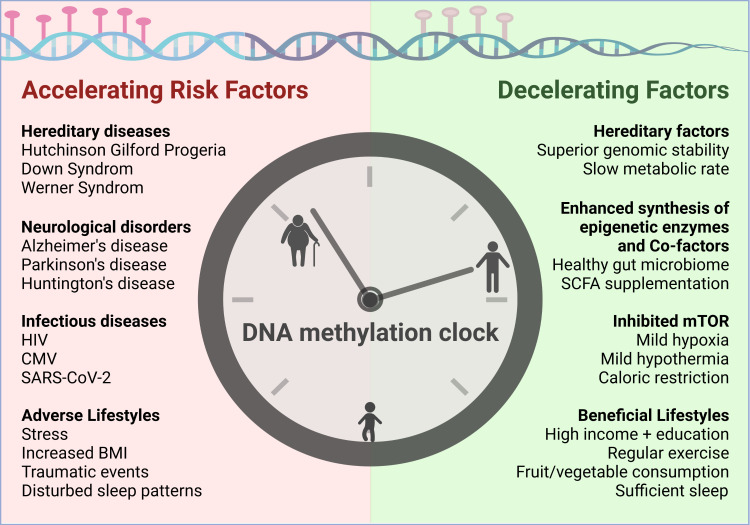
Factors that either negatively or positively affect epigenetic aging. Epigenetic aging as determined with DNAm clocks is accelerated in consequence of several diseases and further negatively affected by some concomitant conditions of certain lifestyles. On the other hand, there are some hereditary and environmental factors that are associated with slowed epigenetic aging.

The first epigenetic clocks were supposed to accurately predict the chronological age of individuals. However, many insights can be gained when a discrepancy between estimated and actual age occurs. Indeed, in several cohorts higher age estimates have been linked with increased mortality risk [167,227,228]. Meta-analyses concluded that a 5-year higher age estimate based on the two most widely applied epigenetic clocks by Hannum and Horvath was associated with an 8 to 15% increased mortality risk [229]. Based on DNAm PhenoAge, which was engineered to consider a variety of aging-related phenotypes, many factors that are either positively (e.g., measures of inflammation, glucose metabolism, overweight, systolic blood pressure, and smoking) or negatively (e.g., levels of education, income, exercise, indicators for fruit/vegetable consumption, and HDL cholesterol) correlated with epigenetic age were revealed [89]. Similar correlations were observed for the seven DNAm-based estimators of plasma proteins used for the GrimAge clock [86]. Figure 3 gives an overview on risk factors for accelerated aging which are outlined in detail in the following section before various intervention strategies are discussed.

### 5.1. Diseases Negatively Impacting Biological Age

Pathologies that are known to accelerate biological aging such as Down syndrome [230], Werner’s syndrome [231], and Hutchinson Gilford Progeria Syndrome [232] have been reported to be accompanied with epigenetic age acceleration, thus supporting the physiological relevance of DNAm-based epigenetic clocks. There are a great number of pre-existing conditions that are associated with an acceleration of epigenetic aging. Neurodegenerative diseases such as Alzheimer’s disease [156], Parkinson’s disease [233] and Huntington’s disease [234] are all associated with accelerated epigenetic age. However, due to the genetic contribution to the pathogenesis of these disorders, it is hard to distinguish cause and effect with respect to the differential methylation patterns underlying the age acceleration. For example, in Alzheimer’s disease, some DNA methylation changes precede the onset of pathological conditions as discussed before [158]. Results are more straightforward when it comes to acquired infectious diseases. Chronic infections triggered by either the human immunodeficiency virus (HIV) [235] or cytomegalovirus (CMV) [236] have been reported to be associated with accelerated epigenetic aging and thus can be considered definite risk factors. Notably, evidence for an epigenetic age acceleration was also proposed after a transient infection with SARS-CoV-2 (COVID-19), though it was only traceable in younger patients [237]. However, since long-term effects of COVID-19 are still to be determined it is not clear whether this impact on the DNA methylation landscape will persist and in turn poses an actual risk factor for a decreased life expectancy.

### 5.2. Lifestyles Negatively Impacting Biological Age

The impact of trauma, stress, insomnia, and lifestyles that are generally associated with these conditions on epigenetic age acceleration is still under debate. In 2017, a meta-analysis of nine cohorts concluded that childhood trauma and severity of posttraumatic stress disorder (PTSD) demonstrate significant associations with accelerated epigenetic aging [238]. However, no significant effects of childhood or lifetime trauma were found when evaluating across all studies. For childhood trauma, associations with epigenetic age acceleration were only observed when using studies that assessed trauma based on the “Childhood Trauma Questionnaire” instead of the “Traumatic Life Events Questionnaire”. Moreover, associations were generally only observed when applying the Hannums clock and not seen for the Horvath clock. Yet, one of the underlying studies employing the Horvath clock might hint at factors mediating the adverse effects of trauma and stress on aging. Indeed, a high number of CpG sites of the Horvath’s clock are located in glucocorticoid response elements and administration of synthetic glucocorticoid dexamethasone shown to affect DNA methylation and transcription in many of the genes neighboring CpGs of the Horvath clock [239]. In line with that, greater diurnal cortisol levels were linked with epigenetic age acceleration in adolescent girls, which in turn was associated with an altered neural structure [240]. In a later longitudinal study of parents and children also utilizing the Horvath clock, sexual abuse during childhood was associated with a 3.41 years higher epigenetic age while other psychosocial adversities such as parental long-term illness or death, parental separation or absence of parents, or hampered maternal bonding did not result in epigenetic age acceleration [241]. The surprisingly distinct effects of these stressors might underlie the inability to detect significant associations across multiple studies.

Still, evidence accumulates that lifetime stress negatively impacts epigenetic aging. In a study assessing the effect of Major Depressive Disorder (MDD) on DNA methylation in blood and brain tissue, the presence of childhood trauma enhanced the correlation of diagnosed depression and higher epigenetic age [242]. Consequently, the authors hypothesized that childhood trauma leaves epigenetic “scars”’ that impact MDD later in life. Symptoms of depressive disorders include sleep disturbances, which themselves have been linked to epigenetic age acceleration even when results were adjusted for depressive symptoms [243]. In line with that, lifestyles that are associated with disturbed sleep patterns, such as long term shift work, have also been linked with increased epigenetic age [244]. Specifically, the respective study reports a correlation of night shift work and hypomethylation in the *ZFHX3* gene, which is implicated in the circadian rhythm. Before, Cedernaes et al. showed that a single night without sleep can already result in methylation changes in the key circadian clock genes *BMAL1, CLOCK, CRY1*, and *PER1* [245]. In sleep deprived individuals, serum levels of the stress hormone cortisol were decreased while plasma glucose concentrations were increased, hinting at the interconnectedness of stress, metabolism, the circadian clock and epigenetic aging.

Interestingly, evidence accumulates that effects of stressors on epigenetic aging are severely dependent on the coping strategies and resilience to stress. When DNA methylation in blood was analyzed in 96 male soldiers evaluated for exposure to combat trauma and the presence of post-traumatic stress disorder (PTSD), it was found that trauma significantly accelerated epigenetic ageing but surprisingly, the development of PTSD symptoms was inversely associated with telomere length and epigenetic age [246]. Later, similar findings were ascertained in a cohort of 160 veterans where current antidepressant use was also related to relatively less epigenetic aging [247]. In another study on combat-exposed veterans, epigenetic age acceleration did not differ between subjects with and without PTSD [248]. However, in veterans with PTSD epigenetic age acceleration was increased in individuals that scored high in the Connor-Davidson Resilience Scale. This correlation was confirmed in another cohort of trauma-exposed civilians, suggesting that high resilience comes at the expense of accelerated epigenetic aging [248]. Together, these results suggest that traumatic events contribute to accelerated epigenetic aging and that superficially effective coping of stress unfortunately even exacerbates this effect.

While a longitudinal study in black, middle-aged women concluded that the negative correlation between income and epigenetic age is mostly attributable to the stress caused by financial pressure and not due to health-related behavioral patterns of diet, exercise, smoking, or alcohol consumption [249], many studies suggest an association of an increased BMI and accelerated epigenetic aging [250,251,252,253]. The negative effects of a high fat diet and increased body weight on epigenetic aging could be replicated in a mice study employing BXD sibling strains that exhibit a wide variation in life expectancy [254]. Body weight was shown to be predictive of strain longevity and amongst many epigenetic alterations associated with differentially methylation in the FTO gene, that is playing a key role in energy homeostasis. The availability of these mouse models facilitates ongoing investigations in underlying mechanisms, which tend to be obscured in human studies characterized by an obviously higher variability of genetic background and environmental conditions.

## 6. Epigenetic Therapy and Intervention to Extend Life Span

The main approaches that are utilized to extend life span to date can be categorized into three: alteration of the environment, drug administration, and genetic intervention [255]. All three approaches have been shown to impact epigenetic mechanisms and/or related metabolic activity [256]. We outlined here that biological age is linked to distinct epigenetic alterations and characterized by a specific epigenetic landscape thus allowing for the use of respective patterns as a highly accurate biomarker of age. As epigenetic alterations are reversible, epigenetic rejuvenation may turn out to be an important lever to slow down or even reverse organismal aging.

One obvious readout for successful intervention of age progression is an increased life span. However, the benefits of such interventions may not be restricted to or manifested in total life span, but rather reflected in a prolonged healthy life span, the health span. Frailty, for instance, is elevated in older animals [257]. However, interventions that attenuate such frailty in old age can improve several parameters for example reducing tremor and gait, diminishing hearing loss and improving breathing [258]. A new work using sophisticated and rigorous analysis of automated cage phenotyping systems has provided a high resolution overview of physiological changes throughout life in female mice [259]. This tool and its resulting data could be used for measuring and evaluating changes in these so-called aging benchmarks in response to therapeutic interventions for aging.

### 6.1. Environmental and Diet Interventions Targeting Epigenetic Mechanisms

A study in 2019 demonstrated that the epigenetic age of transplanted human hematopoietic stem cells is unaffected by the altered conditions in a recipient that greatly differs in age, even 17 years after transplantation [260]. This prompted the authors to suggest that the epigenetic age is cell-intrinsic and not modulated by extracellular stimuli in vivo. In contrast, a more recent preprint reports that treatment of old rats with plasma fractions from young rats significantly reduces the epigenetic ages measured in blood [261]. This effect on epigenetic age was paralleled by reduced cellular senescence in vital organs and improved functional parameters, thus challenging the view of epigenetic age as a feature immutable by extracellular stimuli.

In general, epigenetic mechanisms are crucial to enable organisms to cope with a changing environment and thus are susceptible for environmental interventions. Accordingly, experimentally shifting certain environmental parameters can impact epigenetic mechanisms and thus also affect aging. For example, the modulation of temperature has been shown to impact life span particularly in ectotherms. In drosophila, shifting the temperature from 25 °C to 18 °C attenuates the age-associated increase in histone 4 acetylation and extends the life span of the flies [33]. In humans, acclimatization to high-altitude hypoxia is accompanied by epigenetic changes [262]. Hypoxia-associated changes are suggested to be directly induced by oxygen-dependent TET enzymes [263] or mediated by Hif1ɑ [264] and known to be involved in cancer development [265]. However, hypoxia was also shown to slow down epigenetic aging at least in vitro [266]. This effect might be elicited by the inhibitory effects of hypoxia on mTOR activity [267], which regulates cell growth and survival.

Perhaps the most robust environmental intervention used to extend life span is caloric restriction [268], which was demonstrated to regulate both DNA methylation and histone modifications [269]. Caloric restriction attenuates age-related methylation changes and decreases epigenetic age in diverse organisms. For example, when long-term exposed to 40% reduced calorie intake mice with an average chronological age of 2.8 years exhibit a DNAm based epigenetic age of 0.8 years [270]. In rhesus monkeys with an average chronologic age of 27 years, caloric restriction of 30% resulted in 7 years younger epigenetic age [270]. Moreover, short-term exposure to caloric restriction for only four weeks was sufficient to partially ameliorate age-associated alterations in promoter methylation in aged rats [271]. Caloric restriction was shown to attenuate the age-related decline in *DNMT1, DNMT3B, TET1*, and *TET3* gene expression in male C57BL/6 mice, which might represent the main underlying mechanism to delay age-related methylation changes and thereby extending life span [272]. However, the effects of caloric restriction are by no means universal as for example, many strains of mice show no increased life span or even shortened life span upon reduced caloric intake [273].

Yet, as metabolism and aging are known to be highly interrelated, much research on interventions is focused on this particular lever [274]. Additionally, intermittent fasting and an altered diet with respect to certain nutrients and supplements emerged as alternatives to calorie restriction for humans. The underlying mechanisms of diet interventions involve metabolic signaling pathways such as insulin/IGF1, mTOR, AMPK, sirtuin and FOXO pathways, which are recently known to interact with epigenetic mechanisms [275]. For example, mTOR signaling governs serine and one-carbon metabolism which provide SAM as methyl group donor for DNA methylation [276], while some sirtuins not only act as signaling proteins but also as histone deacetylases. As such, SIRT1 expression was shown to be implicated in calorie restriction induced life span extension [277]. Indeed, dietary intervention in rats resulted in increased SIRT1 expression and reduction of H4K16ac [278]. Interestingly, the levels of histone 3 modifications such as H3K9, H3K27, and H3K56 were increased [278]. Similarly, caloric restriction in old mice resulted in increased liver SIRT1 activation and increased H3K9/K14ac and H3K27ac in circadian-regulated genes [279]. Notably, malate and fumarate supplementation was shown to increase life span in worms and the authors speculate that such additions can impact acetyl-CoA and NAD+ levels, which in turn would affect histone acetylation levels [280].

In general, main nutrients such as lipids, glucose and amino acids influence the longevity of organisms by a variety of signaling pathways. Here, we mention only those pathways that involve epigenetic modifications. For example, lipids were demonstrated to provide a major carbon source for histone acetylation [281] and fatty acid elongase 2 (ELOVL2), one of the most strongly age-correlated genes according to DNAm based epigenetic clocks, is also implicated in lipid metabolism [87]. Profiling caloric restriction induced changes in DNA methylation, gene expression and lipidomics Hahn et al. showed that caloric restriction results in delayed age-related methylation changes, methylation dependent downregulation of genes involved in lipid metabolism, as well as a shift in the lipid profile towards lower triglyceride content and shorter fatty acid chains [282]. Moreover, mono-unsaturated fatty acids were shown to extend the life span in C. elegans mediated by H3K4me3 modifiers [283]. High glucose levels negatively affect lifespan and induce age-related maladies such as diabetes. Accordingly, glucose restriction can be expected to extend life span as suggested from studies in human fibroblasts that demonstrate glucose restriction-induced DNA methylation changes and histone modifications targeting for instance *hTERT* and *p16* expression [284]. As stated before, a link between glucose metabolism and epigenetic aging was also found in drosophila, where the age-associated epigenetic drift of a repressive histone mark results in a reduction of glycolytic gene expression [65]. Another study in drosophila found that an imbalance in dietary amino acids especially of essential amino acids affects life span [285]. Accordingly, a methionine-deficient diet was sufficient to increase maximal life span and health span in mice [286]. Methionine serves as a precursor of SAM thus fluctuations of this essential amino acid can influence DNA methylation. However, studies examining the epigenetic mechanism underlying the life span prolonging effect of methionine restriction are scarce [287].

Accumulating evidence suggests that a polyamine-rich diet can also extend lifespan while decreasing the risk for colon cancer as shown in mice [288]. SAM and putrescine are substrates for polyamine synthesis. Due to the additional intake of polyamines, more SAM is available as a substrate for DNA methylation, thereby counteracting the age-associated global hypomethylation [289]. Additionally contributing to elevated SAM levels, supplementation with folic acid and vitamin B12 in a clinical trial was shown to decrease the epigenetic age in women with a genetically reduced activity of methylenetetrahydrofolate reductase which is implicated in the one-carbon metabolism generating SAM [290].

First attempts to implement the accumulated knowledge on environmental and diet interventions to slow aging in humans have recently been undertaken. In a randomized clinical trial in 50–72 year old men, an eight-week treatment program comprising an optimized diet including supplemental probiotics as well as phytonutrients, sufficient sleep, regular exercise and stress release guidance was tested on its potential to slow down or even reverse biological age [291]. Indeed, the treatment program was associated with an average decrease of epigenetic age of 3.23 years based on the Horvath clock. However, of 18 participants in the treatment group only eight actually experienced a reduction of the epigenetic age, hence the study also emphasizes the high variability in humans and therefore the limited generalizability of any measure.

### 6.2. Pharmacological Intervention Targeting or Impacting Epigenetic Mechanisms

Since the discovery of Sir2 as a longevity factor in yeast [292], sirtuins are discussed as potential targets for aging intervention strategies [293]. For example, the SIRT1 activators SRT2140 and SRT1720 have been shown to improve health and extend life span in aging mice [294,295]. Additionally, replenishing NAD+, a cofactor needed for the activity of sirtuins, increased the life span of mice in part by inducing the mitochondrial unfolded protein response and consequently attenuating stem cell senescence [296].

Interestingly, not only decreasing histone acetylation by enhanced sirtuin activity but also increasing acetylation levels has been demonstrated to have beneficial effects on aging and age-related maladies. For example, two compounds that increase acetyl-CoA levels and consequently acetylation of histone H3K9 attenuated brain aging [75]. Moreover, various molecules that inhibit HDAC/KDAC drew considerable attention in epigenetic therapy for a wide range of maladies including cancer, neurodegeneration, and others [163,221,297,298,299,300,301]. However, whether such inhibitors can robustly extend mammalian life span remains unclear as several studies in model organisms hint at the intricate application with respect to dosage and timing.

Trichostatin A (TSA), a wide range HDAC/KDAC inhibitor was shown to increase the life span of male and female drosophila at 10 µM, however it is noteworthy that the flies in this specific study were remarkably short lived compared to other studies [302]. In contrast, the addition of 40 µM or 400 µM TSA to young adult flies resulted in mild reduction of the life span in males [33]. The addition of valproic acid, a more specific HDAC/KDAC inhibitor, resulted in an increased life span in worms [303]. Interestingly, only lower doses of 3–6 mM produced a positive effect, while dosages above 12 mM reduced life span. Conversely, only the addition of above 20 mM, D-beta-hydroxybutyrate (D-βHB), another HDAC/KDAC inhibitor, increased life span in worms by roughly 20% [304].

Another well-studied HDAC inhibitor is sodium butyrate (SB). The addition of 15 mM and 150 mM SB to young adult drosophila resulted in mild and drastic decrease in life span, respectively [33]. However, addition of various SB concentrations at developmental stages increased the life span of drosophila [305]. SB was also shown to positively impact longevity in mouse models of premature aging. The addition of 4 g/L of SB to Zmpste24−/− mice extended their life span, although the authors add that the addition of 8 g/L was toxic [24]. Interestingly, the SB treated mice display lower senescence burden and improved bone density [24]. Overall, it is apparent that both the dose and timing of administering a HDAC/KDAC inhibitor is essential to successfully increase life span. This concept is further illustrated by Zaho et al. who demonstrated distinct survival outcomes when adding TSA and SB only during development of the flies versus maintaining the treatment also during adulthood [306]. The authors also show that similar TSA and SB treatments have different outcomes in short lived versus longer lived fly strains [306].

As previously mentioned, growing amounts of data imply that the acetylation of numerous non-histone proteins can be impacted by HDAC/KDAC inhibitors [57,307]. For example, SB may potentially impact life span by acting on the metabolic rate [33,224,225] or the gut microbiome, which makes it further difficult to isolate the impact of SB on aging solely via histone acetylation [308]. The same overall general concerns apply to the usage of HAT inhibitors. Adding to the yeast food several HAT inhibitors (epigallocatechin gallate, anacardic acid, garcinol, and curcumin) resulted in prolonged life span [309]. Another HAT inhibitor, NDGA, which inhibits p300, was shown to increase life span in worms [310], mosquitoes [311] and male mice [312]. Interestingly, the polyamine spermidine was also shown to inhibit the general HAT activity in nuclear extracts of yeast, resulting in decreased H4K9ac, H3K14ac and H3K18ac levels during aging and an increased life span [313]. Collectively, it is surprising that opposite inhibitions, namely promoting either hyper or hypo histone acetylation, both lead to increased life span. More work is needed to clarify this topic and uncover common pathways that are impacted by both HDAC and HAT inhibitions, that are yet independent of histone acetylation.

Importantly, a number of other drugs suggested to increase life span impact epigenetic mechanisms [255]. For example, rapamycin, a prominent inhibitor of mTOR, that was shown to increase the median life span of mice by 23-26% [314], does not only cause a corresponding decrease in DNA methylation age [315] but was also reported to directly affect a number of histone marks [316]. Acting through mTOR complex 1 and 2, rapamycin treatment for instance significantly reduces p300 and histone H3 acetylation thereby regulating autophagy and lipogenesis [317,318]. These findings demonstrate that life prolonging effects of rapamycin are, at least partially, mediated via epigenetic alterations.

Metformin, another mTOR signaling inhibitor, is also reported to increase life span in various animal models [319,320]. Originally developed as anti-diabetic drug, metformin is reported to have multiple targets [321]. As one of the targets is AMP-activated kinase, which impacts epigenetics, it is speculated that metformin may impact histone modifications [322]. Indeed, metformin treatment increases histone acetylation, as well as protein acetylation [323] and alters histone marks in different cancer cell lines [324]. Moreover, metformin fosters AMPK-mediated phosphorylation and stabilization of TET2, thereby modifying 5-hydroxymethylcytosine levels and linking diabetes to cancer via an epigenetic mechanism [325]. However, in a longitudinal study employing the DNAm based epigenetic clock of Horvath et al. its effect on epigenetic aging could not be detected in human participants [251]. More work is needed to elucidate whether metformin treatment can attenuate age-associated epigenetic deregulations.

**Figure 4:**
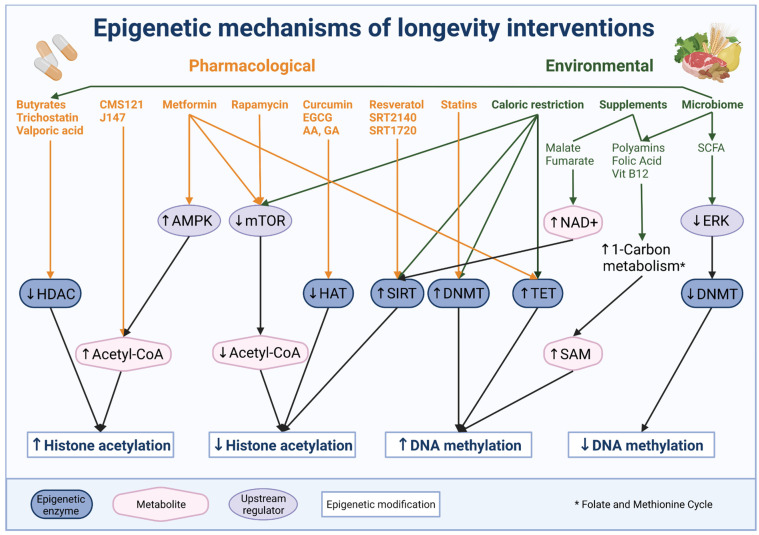
Pharmacological and environmental longevity interventions impacting epigenetic mechanisms. A number of pharmacological substances mediate their beneficial effects on life span via epigenetic enzymes or upstream regulators that influence levels of crucial metabolites. Caloric restriction was demonstrated to positively affect life span acting via similar mechanisms, while some amino acids and vitamins support the generation of metabolites for methylation and acetylation.

Another drug family originally not intended to act on epigenetic aging are statins. Used for decades to lower cholesterol levels in patients with atherosclerotic heart disease, there is evidence that statins also lower DNA methylation through inhibition of DNMTs [326]. Statin-induced epigenetic modifications can result in enhanced expression of genes with anti-atherosclerotic actions and are also reported to prevent silencing of tumor suppressor genes in cancer [326]. The relevance of these mechanisms for the preventive effect of this drug still needs to be assessed. However, in view of these findings, statins might also be considered as epigenetic drugs to extend health span. An overview of pharmacological and environmental interventions and their impact on downstream targets is given below in Figure 4.

### 6.3. Genetic Interventions Targeting Epigenetic Modifiers, Metabolic Linker and Epigenetic Reprogramming

A straightforward approach to extend life span is genetic intervention aiming to attenuate or counteract specific age-associated alterations in the epigenome [255]. Counteracting the age-related decrease of DNA methylation, overexpression of DNMT2 was shown to increase life span in drosophila [327], while decreased global DNA methylation in heterozygous DNMT1-deficient mice negatively affects their health span [328]. However, as age-related epigenetic alterations are complex and locus-specific hypermethylation is equally implicated in aging and aging-associated maladies, global overexpression of DMNTs has not been established as an intervention method.

Targeting histone modifiers is another approach to counteract age-related epigenetic alterations. For example, deleting sas2 in yeast was shown to attenuate the age-associated increase in H4K16ac and extend life span [31]. Similarly, reduction of *chm* attenuates the age-associated increase in H4K12ac, attenuates transcriptional deregulation, and increases life span in drosophila [33]. Conversely, histone deacetylase complex (*HDAC*) yeast mutants display higher H3K18ac levels and demonstrate an extended life span [329]. The functional role of this complex for longevity is suggested to be conserved across species as inactivating *HDAC6* also resulted in an increased life span in C. elegans and drosophila [329]. Moreover, reduction of the HDAC rpd3 also correlates with life extension in flies [330,331]. Nonetheless, it is important to note that such genetic intervention may have a direct impact on non-histone acetylation that might contribute to observed changes in life span [33,57,226].

Overexpression of sirtuins has been proposed to decrease histone acetylation and enhance cellular life span [31,293]. In mice, SIRT6 overexpression resulted in a significantly prolonged life span, but interestingly this effect was only observed in male animals [332]. Brain-specific overexpression of SIRT1 induced delayed aging and a significant life span extension in both male and female mice [333]. However, the robustness of reported effects of sirtuins on life span was challenged in C. elegans and drosophila [334] and more work is needed to confidently link sirtuin activity with attenuation of aging.

Histone methylation has also been targeted for life span extension [335]. Reducing members of the ASH-2 trithorax complex, that regulate the trimethylation of H3K4, can extend life span in worms [336], at least partially via promoting fat accumulation and in particular, via specific enrichment of mono-unsaturated fatty acids [283]. Furthermore, mutating members of the methyltransferase PRC2 such as *E(z)* resulted in decreased H3K27me3 and substantially increased life span in flies [337]. Similarly, a later study showed that overexpression of the demethylases jmjd-1.2 and jmjd-3.1, which regulate H3K27me, is linked with increased life span in worms [338]. This effect was dependent on the mitochondrial unfolded protein response (UPR) thus stressing the link between metabolism, epigenetics and longevity [338].

Conversely however, reduction of the demethylases jmjd-2, set-9/26, mes-2, utx-1, and rbr-2 in C. elegans is reported to increase life span, which was attributed to a prevention of age-dependent loss of the repressive histone marks H3K9me3 and H3K27me3 [339]. This contradiction is well discussed by Han and Brunet [335], who illustrate that the nature of epigenetic alterations and thereof possible interventions can be substantially different or even opposite depending on tissue, sex, species and timing of an intervention.

Further, such interventions might affect targets that are unrelated to histone methylation [335]. Indeed, recent results by Guillermo et al. support this notion. Their study shows that reduction of either the methyltransferase mes-2 or the demethylases jmjd-3.2 and utx-1 results in life span extension, which is intriguing, as they are believed to have opposite regulation on H3K27me3 levels [340]. Even more surprisingly, overexpression of the very same jmjd-3.2 and utx-1 also results in a life span extension [340]. While these confounding results should be further supported and solidified by further studies, the authors also raise the possibility that both classes of enzymes have non-histone substrates [340]. Further studies broadening the impact of targeting methyltransferase or demethylases on the general protein methylome are needed to consolidate this hypothesis [341].

Other genetic interventions to modulate life span target acetyl-CoA metabolism and related histone acetylation. For example, brain specific knockdown of acetyl-CoA synthase (AcCoAS) was demonstrated to increase the life span of female flies and the maximal life span of male flies [77]. Moreover, modest reduction of ATP citrate lysase (ATPCL) resulted in an increased life span of flies [33] while overexpression of AcCoA-synthetase (ACS2) led to a reduction in yeast life span [77]. A recent study by Zhu et al. reports that mitochondrial stress leads to a reduction of citrate and acetyl-CoA levels in worms, ultimately leading to a nuclear accumulation of the nucleosome remodeling and histone deacetylase complex (NuRD), decreased overall histone acetylation and increased life span [342]. Conversely, components of the same complex become diminished in aging humans, as observed in cells from Hutchinson-Gilford progeria syndrome (HGPS) patients and aging fibroblasts [343]. Lastly, recent preliminary data support the notion that overexpressing citrate carrier, and therefore histone acetylation, might improve the differentiation capacity of mesenchymal stem cells during aging [78].

The most recent and promising genetic intervention approaches rely on reprogramming, which has been shown to reset the epigenetic clock [85], telomere length and gene expression profiles [344]. Reprogramming of adult somatic cells into a pluripotent state can be achieved with four transcription factors *OCT4, SOX2, KLF4*, and *MYC* (OSKM) [345]. Single-cell expression profiling by Buganim et al. revealed that this process involves two main phases accompanied by specific epigenetic remodeling processes. An initial phase of stochastic gene expression is followed by a late hierarchical phase activating core pluripotency genes such as *NANOG* and *OCT4* [346]. Epigenetic changes in the early phase involve for example acquisition of the active mark H3K4me2 and loss of the repressive mark H3K27me3 while genes of the later phase are often unmarked. Changes in genome-wide promoter DNA methylation are also a characteristic of the late phase in reprogramming [347,348].

Notably, when reprogramming fibroblasts from HGPS patients, generated iPSCs show no accumulation of progerin, and no epigenetic alterations normally linked to the premature aging phenotype associated with this syndrome [349]. However, upon differentiation of these iPSCs, progerin is restored, consequently reestablishing the aging-associated phenotype. Applying an optimized protocol additionally containing *NANOG* and *LIN28*, Lapasset et al. demonstrated that iPSCs can even be generated from senescent and centenarian cells while fully resetting their telomere length, gene expression profiles, oxidative stress levels, and mitochondrial metabolism in the process [350]. This rejuvenated state was also conserved upon redifferentiation thus inspiring attempts to rejuvenate whole tissues and organisms. Indeed, overexpression of *NANOG* restored the levels of the heterochromatin markers H3K9me3 and H3K27me3 in senescent cells, bringing them back to similar levels as observed in young cells [351]. Importantly, this treatment improved mitochondrial function and autophagy, and restored the number of myogenic progenitors in a LAKI mouse model of progeria [351].

In transgenic mice, transitory systemic induction of OSKM resulted in dedifferentiation of cells, marked by the pluripotency marker *NANOG*, in several organs, thus proving the feasibility of in vivo reprogramming [352]. However, this was associated with the formation of teratomas. Later, the emergence of tumor cells, subsequent weight loss, and mortality upon in vivo reprogramming in transgenic mice was attributed to altered DNA methylation levels [353]. To avoid the adverse effects of chronic OSKM expression, Ocampo et al. confined to partial reprogramming by applying a protocol of short-term cyclic induction of OSKM expression in a mouse model of premature aging [354]. In this way, cellular and physiological features of aging could be attenuated, levels of H3K9me3 and H4K20me restored, and consequently the life span of treated mice extended by approximately 15% [354].

At present, it is hypothesized that partial reprogramming results in a continuously decreasing epigenetic age before cell identity is lost [355]. While a linear decrease in epigenetic age was observed within the first 20 days of fibroblast reprogramming, full pluripotency was only reached after 28 days [356]. This raises hope that the loss of cell identity and the reset of the epigenetic clock can be uncoupled, thus allowing for rejuvenation without increased risk for tumor formation. Indeed, two studies in 2020 seem to prove the feasibility of such an approach. Based on a non-integrative reprogramming approach mediated by application of an mRNA cocktail (*OCT4, SOX2, KLF4, c-MYC, LIN28*, and *NANOG*) for four consecutive days, several attributes of aging could be attenuated across multiple human cell types [357]. Treatment outcomes included resetting of the epigenetic clock, a reduction of inflammation and oxidative stress in chondrocytes, and improved regenerative potential of aged muscle stem cells. This study demonstrated that cellular rejuvenation is generally feasible in naturally aged human cells without compromising their identity or functionality. In the second study only three factors (*OCT4, KLF4* and *SOX2*) were introduced using a regulatable adeno-associated viral vector to improve regeneration and counteract age-related functional decline of neuronal tissues employing the murine eye as a model system [358]. Continuous expression of the reprogramming factors for four weeks was sufficient to reset the DNA methylation profile, promote regeneration after injury and eventually restore vision both in an experimental model of glaucoma and in 12 month old mice experiencing the typical age-related vision impairments [358]. Tet1 and Tet2 were reported to be crucial for these regenerative effects suggesting that they play an essential role in resetting the epigenetic clock and cellular rejuvenation. Of note, omitting the oncogene c-MYC, even continuous expression of the reprogramming factors in mice for up to 18 month did not induce pathological changes [358]. These studies point to the immense potential of epigenetic therapy to recover age-related impairments and expand human health span.

## 7. Outlook

Currently, differential gene expression in young and aged organisms is often analyzed to assess the physiological relevance of the epigenetic modifications discussed above. However, transcriptional consequences of epigenetic changes might manifest only in a small number of cells, while their respective expression profiles can be masked by the excess of transcripts from unaffected cells. As single-cell sequencing and single-cell DNA methylation profiling gain traction, more sensitive studies might soon uncover more underlying mechanisms and affected pathways thereby also enlightening the physiological relevance of age-associated epigenetic changes. Indeed, in recent years, we observe publications of more complex data sets such as single-cell epigenetic and transcriptomic profiling [26,359,360,361] and first single-cell and multi-omics studies during ageing are already conducted [362,363]. Understanding how single cells are epigenetically altered during aging is a key to detect earlier changes in a subset of cells, which may enable us to embrace a more preventive approach to delay aging. Respective bioinformatic tools to study epigenetic age at single-cell resolution were recently developed [364].

Advances in the field of single-cell proteomics will yield novel data linking age-associated epigenetic changes with protein abundances. Generally, multi-modal age predictors that integrate multiple data types, will certainly provide deeper insights into age related alterations and the link between cause and effect. Moreover, integrating data from epigenetic marks, transcription and physiological condition of animals throughout aging can facilitate discoveries of new targets for interventions. Essentially, this may lead to personalized therapies for individuals based on their specific overall epigenetic alterations. A first step along this path is the utilization of automated high throughput techniques for drug screening. For example, using the recently developed worm boot by the Kaeberlein group may enable the parallel testing of an enormous number of compounds in worms, thereby facilitating the discovery of previously unrecognized substances for life and health span extension [365].

Alternative approaches to assess the consequences of epigenetic alterations might complement the search for intervention strategies. Nowadays, refined imaging methods allow for high-contrast visualization of chromatin dynamics during biological processes such as aging. Epigenomic alterations at telomeres and centromeres as well as attrition of ribosomal DNA repeats were identified in premature aging models [366] and might soon be investigated for natural aging.

Within the growing amount of data in the field, we envision greater involvement of deep learning approaches for downstream analysis, frequently designated as Artificial Intelligence (AI). In the recent past, several aging clocks have been developed that already utilize deep learning approaches instead of linear regression methods that used to be applied for the classical epigenetic clocks. The first aging clock relying on deep neural networks was introduced by Putin et al. in 2016 [367]. Trained on data from common blood biochemistry and cell count tests of more than 60,000 samples originating from diverse populations, the clock demonstrated high predictive accuracy in individual populations and moreover revealed ethnic differences in biological aging [368]. This year, the first epigenetic clock using a deep neural network model, termed DeepMAge, was presented [369]. The model was trained on around 5000 blood DNA methylation profiles across 17 studies and provides higher accuracy in age prediction compared to the Horvath clock, potentially initiating a generation change for epigenetic clocks.

Additionally, transcriptomic data, physical activity and image data were used to develop age predictors. Moreover, neural networks can be used to generate synthetic molecular and patient data facilitating in silico biomarker development and drug discovery, which is already utilized in pharmaceutical research by large companies such as Longevity Vision Fund, Juvenescence.AI, and Life Biosciences [370]. The economic interest of pharmaceutical companies may substantially accelerate scientific progress in this field.

However, more intriguingly from the epigenetic point of view is the mathematical model proposed by Galkin et al. that can be applied to predict the biological age of humans based on their gut microbiome [371]. Although the trained deep neural network demonstrates a mean absolute error of 5.91 years and thereby obviously performs less accurately compared to DeepMAge, it is of major relevance for the field. On the one hand, it identifies specific taxa of microbes as potential aging biomarkers, and on the other hand, it strongly suggests a link between the microbiome and epigenetic pathways. Other groups applied deep learning approaches to decipher these interrelations under age-related pathological conditions such as diabetes [372] and neurological disorders [373].

Apparently, the microbiome is linked to epigenetics by gut metabolites such as vitamins and short-chain fatty acids (SCFAs) [374]. Folate, vitamin B12, betaine, and choline are indirectly involved in DNA methylation, as they are implicated in the synthesis of SAM via the folate and methionine cycle [375]. The availability of these vitamins is in part regulated by specific gut bacteria such as *Lactobacillus* and *Bifidobacteria* [376]. SCFAs such as butyrate were shown to influence DNA methylation by suppressing ERK activation and down-regulating DNMT1 [377]. Moreover, in multiple host tissues global histone acetylation and methylation levels are affected by small-molecule metabolites and SCFAs in particular [378]. The intake depends on the diet, but is also heavily influenced by microbial colonization as several microbes, for instance, can produce butyrate [379]. Supernatants of three butyrate-synthesizing bacterial strains, namely *Megasphaera massiliensis MRx0029, Roseburia intestinalis MRx0071*, and *Bariatricus massiliensis MRx1342*, were reported to inhibit HDAC enzymes in human cells, thereby strengthening the suggested link between microbiome and epigenetic landscape [380]. Intriguingly, transfer of the natural gut microbiome of young African turquoise killifish to middle-aged individuals counteracted the age-related decline in microbial diversity, induced beneficial systemic effects, and prolonged the life span of treated fish [381]. The fact that some gut microbiome features are associated with longevity also in humans [382] points to an exciting new avenue for interventions that target metabolism and epigenetics to extend health span.
